# Low-light image enhancement via adaptive frequency decomposition network

**DOI:** 10.1038/s41598-023-40899-8

**Published:** 2023-08-29

**Authors:** Xiwen Liang, Xiaoyan Chen, Keying Ren, Xia Miao, Zhihui Chen, Yutao Jin

**Affiliations:** School of Electronic Information and Automation, Tianjin University of Science and Technology, Tianjin, 300222 China

**Keywords:** Computer science, Software, Information technology, Electrical and electronic engineering

## Abstract

Images captured in low light conditions suffer from low visibility, blurred details and strong noise, resulting in unpleasant visual appearance and poor performance of high level visual tasks. To address these problems, existing approaches have attempted to enhance the visibility of low-light images using convolutional neural networks (CNN). However, due to the insufficient consideration of the characteristics of the information of different frequency layers in the image, most of them yield blurry details and amplified noise. In this work, to fully extract and utilize these information, we proposed a novel Adaptive Frequency Decomposition Network (AFDNet) for low-light image enhancement. An Adaptive Frequency Decomposition (AFD) module is designed to adaptively extract low and high frequency information of different granularities. Specifically, the low-frequency information is employed for contrast enhancement and noise suppression in low-scale space and high-frequency information is for detail restoration in high-scale space. Meanwhile, a new frequency loss function are proposed to guarantee AFDNet’s recovery capability for different frequency information. Extensive experiments on various publicly available datasets show that AFDNet outperforms the existing state-of-the-art methods both quantitatively and visually. In addition, our results showed that the performance of the face detection can be effectively improved by using AFDNet as pre-processing.

## Introduction

In order to convert a given low-light image into a high-quality image with appropriate brightness, some low-light image enhancement methods have been proposed and achieved remarkable result. In general, low-light image enhancement methods can be divided into two branches: traditional methods^1–11^ and CNN-based methods^12–24^. Traditional methods mainly refer to histogram equalization (HE)-based and Retinex-based methods. HE-based methods^1–6^ stretch the dynamic range of the image by manipulating the corresponding histogram, and increase the local adaptability by adding constraints and side information. However, due to the lack of recognition and utilization of semantic information in the enhancement process, most HE-based methods are still not flexible enough to adjust the visual properties of local regions. When processing low-light images with complex information, problems such as color shift and amplification noise are prone to occur.

The Retinex-based methods are on the foundation of Retinex theory^25^ that the color of an object is not determined by the composition of light but from the object itself, and decomposed the image into reflection and illumination components. By further processing and combining, it can achieve the enhanced results. In the past decades, various priors and constraints^7–11^ is proposed to remove noise and recover high frequency detail information. These methods have achieved impressive results in stretching image contrast and denoising. However, these assumptions or models are established under specific conditions and cannot well deal with natural images formed under various complex imaging conditions. Therefore, Retinex-based methods have certain limitations in applicability.

**Figure 1:**
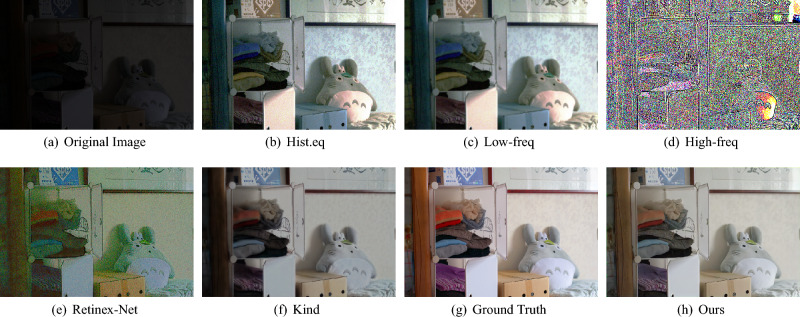
Visual comparisons on a typical low-light image. Given a low-light RGB image (**a**), the result obtained by using the existing algorithm is (**b,e,f**). In comparison, the enhanced images obtained with AFDNet are closer to the ground truth (**g**) and have good perceptual quality. We apply a Gaussian filter to decompose (**b**) to obtain the low-frequency information (**c**) and the high-frequency information (**d**).

In recent years, convolutional neural networks (CNN) have been widely used in various fields of image processing^15,26,27^, including low-light image enhancement^12–24^. Due to its excellent performance and flexibility, CNN-based methods have received much attention from researchers. Specifically, Lore et al.^14^ used a deep autoencoder called Low-Light Net (LLNet) for simultaneous contrast enhancement and denoising. Inspired by this approach, more complex CNN architectures^22–24^ were used for low-light image enhancement. In^16,18,19^, Retinex structures are fused into effective deep network design to absorb the advantages of Retinex-based methods (i.e. good priori structure) and learning-based methods (i.e. the useful prior information extracted from large-scale datasets). Very recently, Liu et al.^28^ built a Retinex inspired unrolling framework(RUAS) with architecture search. Ma et al.^29^ develop a Self-Calibrated Illumination (SCI) learning framework for fast, flexible and frobust low-light image enhancement. There methods can better solve the degradation problem in the image, but there are still color distortion, noise amplification and detail lost.

In the field of image processing, recent works^30–32^ have introduced frequency decomposition networks, achieving impressive results. Specifically, Xu et al.^33^ proposed a two-stage enhancement method for frequency-based decomposition and enhancement. Xu et al.^21^ explored the frequency distributions of the feature maps extracted from different layers of a CNN model and try to seek the best representation for the illumination and edge information.

**Figure 2:**
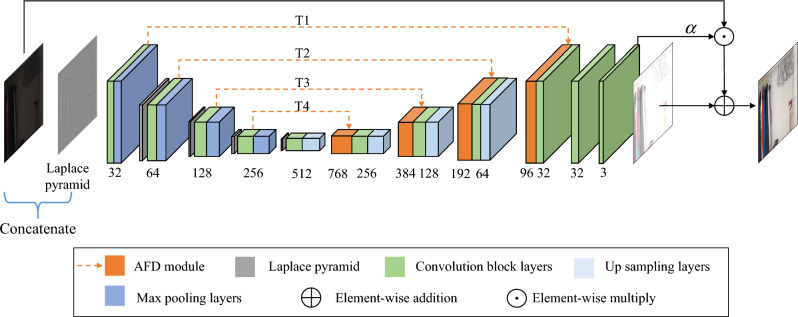
The overall architecture of AFDNet. The input is decomposed into five-scale Laplacian pyramids by decomposition, and feature fusion is performed by channel splicing in the encoding branch. In the decoding branch, the AFD model is used instead of the traditional skip connection method to gradually fuse the frequency features extracted by the encoding branch with the features extracted by other branches. The residual map output by the network is added to the result of multiplying the input image by $\alpha$ to obtain the image enhancement result, where $\alpha$ is a learnable parameter.

As shown in Fig. 1, given a low-light RGB image (Fig. 1a), the results obtained using the existing algorithm are (Fig. 1b,e,f), and it is obvious that the obtained results have the problems of color distortion (Fig. 1e), noise amplification (Fig. 1b), and detail blurring (Fig. 1f). In comparison, the enhanced images obtained with our method (Fig. 1h) are closer to the ground truth (Fig. 1g) and have good perceptual quality. To investigate the characteristics of the low-frequency and high-frequency components, We use Gaussian filtering to decompose the enhanced result (Fig. 1b) into two sub-images with low and high frequency layer (Fig. 1c,d). It is obvious that the low-frequency layer contains mainly luminance and color information, while the high-frequency layer contains rich noise and detail information. Then, we further analyze the frequency distributions of the feature maps extracted from different U-Net^34^ layers (hierarchical features). We found that in the low-scale space of U-Net mainly includes low-frequency information, and in the high-scale space mainly includes high-frequency information. To fully extract and utilize the frequency information of different layers, we propose a novel Adaptive Frequency Decomposition Network (AFDNet). Specifically, a adaptive frequency decomposition (AFD) module is introduced to mine frequency information in appropriate network layers, extracting low-frequency information from low-scale space (i.e. T3 and T4 layers in Fig. 2) and exploiting high-frequency information from high-scale space (i.e. T1 and T2 layers in Fig. 2). Verified by extensive experiments, AFDNet achieves more robust result for all degraded images. In general, the contributions are as follows:

- Design a novel Adaptive Frequency Decomposition Network (AFDNet) to extract frequency information from coarse to fine. Adaptive Frequency Decomposition (AFD) module is the core of AFDNet, which connects shallow features and deep features to extract low-frequency and high-frequency information for detail recovery and noise suppression. Through end-to-end training, both low-frequency and high-frequency information of the image are effectively recovered.
- The idea of self-regularization is introduced to both the Laplacian pyramid and the Generative Adversarial Network(GAN) to enhance detail recovery ability.
- A multi-term loss function composed of frequency, content, adversarial, mutual consistency and total variation terms, allowing an efficient image quality estimation.
- We conduct extensive experiments on six public datasets to demonstrate the superiority of our model in both qualitative and quantitative metrics.

The rest of this paper is organized as follows. “Related work” section briefly reviews related work. “Methodology” section introduces the proposed AFDNet for low-light image enhancement. Experimental results and concluding remarks are given in “Experiments” and “Conclusions” sections, respectively.

## Related work

### HE-based methods

HE^1^ was proposed to increase the contrast of an image by expanding the dynamic range of the entire image. In the beginning, the initial HE cannot solve complex problems such as severe noise and insufficient lighting in low-light images. Many researchers have made various improvements to the original HE to improve its performance. Lee et al.^2^ proposed to optimize contrast enhancement according to a 2D histogram hierarchical difference approach, and Wu et al.^5^ adaptively controlled contrast gain according to the intensity and potential visual importance of pixels.Subsequently, diversified constraints^3,4^ are proposed to improve the overall visual quality. In order to improve the local adaptive ability, some methods^5,6^ adopt a finer-grained way to better adjust the histogram. However, the HE-based method is not flexible enough for local area adjustment, resulting in poor local appearance such as underexposure/over-exposure and noise amplification.

### Retinex-based methods

The Retinex theory^25^ decomposes an image into two parts: the reflectance and the illumination component, where the reflectance component is consistent under any lighting condition. Usually we can estimate the illumination component from the original image, and then try to remove or reduce it to achieve the purpose of low-light image enhancement. Based on Retinex theory, a series of methods are proposed. Single-scale Retinex (SSR)^7^ enhanced image edge information by filtering out low-frequency information and retaining high-frequency information. In order to solve the blurring of local details and the halo at strong edges after SSR processing, Multi-scale Retinex (MSR)^8^ fused SSRs of different scales. Multi-scale Retinex Band Color Restoration(MSRCR)^9^ added a color restoration factor to MSR to compensate for color distortion defects caused by contrast enhancement in local areas of the image. In recent years, Fu et al.^35^ proposed a weighted variational model in which more reflexive details are preserved by adding a better a priori representation for the regularization term. Li et al.^36^ extended the traditional Retinex model to a robust model with an explicit noise term and made the first attempt to estimate the noise map of this model by minimizing the alternating directions. Fu et al.^10^ proposed a straightforward and effective fusion-based low-light image enhancement method. Xu et al.^11^ designed a local derivative filter to extract structure and texture maps for regularizing the enhancement of illumination and reflectance layers. However, the hand-made constraints make it difficult to accurately decompose low-light images into reflectance and the illumination component, resulting in unnatural visual effects.

### CNN-based methods

With the continuous development of deep learning technology, researchers have found that low-light image enhancement using deep learning has good flexibility and performance which gradually become the mainstream direction of low-light image enhancement. Zhu et al.^24^ proposed a two-stage edge-enhanced multiple-exposure fusion network for image enhancement. Lv et al.^17^ proposed a multi-branch low-light image enhancement network (MBLLEN) to extract a large number of features from multiple branches and fuse them into the final enhanced image. Shen et al.^15^ used CNN to simulate the traditional multi-scale Retinex low-light image enhancement process, and established an end-to-end model to learn the mapping from low-light images to normal-light images. Based on the Retinex theory, Zhang et al.^16^ built a fully convolutional network to complete the decomposition and enhancement operations, and introduced the BM3D^37^ denoising module to remove the noise in the reflection image. Zhang et al.^18^ designed a Kind network and constructed a fully convolutional network based on Retinex theory. Its network framework consists of three sub-networks, including: decomposition network, illumination map adjustment network and reflection map restoration network. It can better solve the problem of image degradation and noise in reflection map. Subsequently, the Kind++^19^ network was developed to illuminate the dark areas while also removing hidden artifacts and suppressing noise. In addition, the researchers have proposed some unsupervised deep network models for low-light image enhancement. Jiang et al.^13^ proposed an unsupervised generative adversarial network EnlightenGAN, which gains remarkable performance by imposing (i) gobal-local discriminator to balance global and local image enhancement; (ii) self-feature preservation loss and self-regularization attention mechanism Realize the idea of self-regularization. Guo et al.^12^ proposed a zero-reference learning framework Zero-DCE, which learns the mapping relationship between low-light images and curve parameters through a set of effective non-reference loss functions, and enhances image brightness and contrast in an iterative manner. Furthermore, Li et al.^20^ provided an accelerated version of Zero-DCE++, which significantly improves the efficiency of the computation and keeps the performance almost the same.

The aforementioned methods have a good performance in improving low-light image quality. However, due to the importance of frequency information to image reconstruction is not fully considered, most methods still cannot simultaneously solve all problems such as: noise amplification, color distortion, and fuzzy details. In this work, we explore the characteristics of low and high frequency information and analyze the frequency distribution of the feature maps extracted from different U-Net layers. Based on this, we designed an AFDNet to extract the illumination and edge features in different network layers, enabling the enhancement model to achieve satisfactory results. We design a novel frequency loss function to constrain the network to better recover the information of different frequency layers. Figure 2 shows the network framework of the proposed AFDNet.

## Methodology

### Laplacian pyramid

Inspired by^38^, we introduce a Laplacian pyramid in the image space to provide multi-scale residual information for the network. Specifically, the coarsest level of the Laplacian pyramid guides the network to adjust the global illumination, and the finer pyramid levels of the Laplacian pyramid force the correlation network to recover image local details. The input image ${I_1}$ is first decomposed into a five-level Laplacian pyramid, which can be formulated as follows:

1 $$\begin{aligned} I_{k+1}= & {} f_\downarrow (I_k), \end{aligned}$$

2 $$\begin{aligned} L_k= & {} I_k-f_\uparrow (L_{k+1}), \end{aligned}$$

where $k\in (1,2,3,4)$ represents the level of the Laplacian pyramid. $f_{\downarrow }$and $f_{\uparrow }$ are both down-sampling and up-sampling by bilinear interpolation. Noted that ${L_5}={I_5}$, which is acquired by downsampling the original image to the 1/16 scale. The Laplacian Pyramid has the following advantages: (1) Provide multi-scale Laplacian residual image features to guide the encoder-decoder architecture to recover image details. (2) Provide richer and more realistic texture features at multiple scales. (3) Add higher-level and more abstract features to improve the robustness of the network.

### AFD module

**Figure 3:**
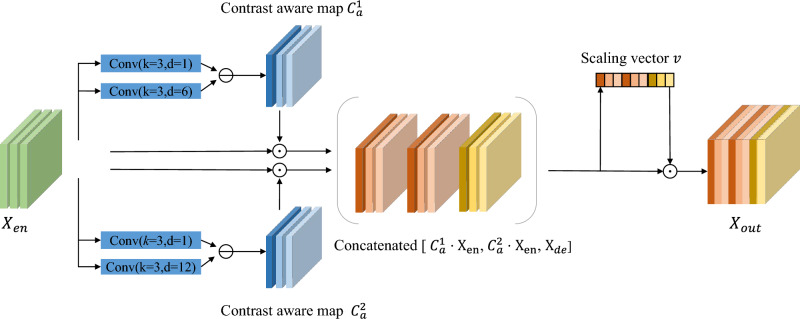
Overview of the proposed AFD module.

In order to obtain more frequency-aware information for image enhancement, we propose AFD module to connect encoding and decoding, and its block diagram is shown in Fig. 3. The adaptive frequency decomposition process can be written as:

3 $$\begin{aligned} C_a^i= & {} \delta (f_{d_1^i}^i(x_{en}) - f_{d_2^i}^i(x_{en})), \end{aligned}$$

4 $$\begin{aligned} high_f= & {} [C_a^1 \cdot x_{en},\cdots , C_a^i \cdot x_{en}], \end{aligned}$$

5 $$\begin{aligned} low_f= & {} [(\alpha ^1-C_a^1) \cdot x_{en},\cdots , (\alpha ^i-C_a^i) \cdot x_{en}], \end{aligned}$$

where $i\in (1,2)$, $C_a^i$ represents the contrast-aware attention map of different branches. $f_{d_1^i}^i(\cdot )$ and $f_{d_2^2}^i(\cdot )$ represent convolution operations with a kernel size of 3$\times$3, the dilation rates are $d_1^i$ and $d_2^i$, respectively. $\delta (\cdot )$ is the linear activation function Leakyrelu. Inspired by^33^, $C_a^i$ represents pixel-level contrast information, where pixels of high contrast are considered to be the high frequency layer of the image. The high-frequency information can be extracted by multiplying $C_a^i$ by the input feature $x_{en}$, and the low-frequency information can also be extracted by multiplying $(\alpha ^i-C_a^i)$ and $x_{en}$. $\alpha ^i$ represents a learnable parameter that controls the intensity of low-frequency information. The frequency-aware information of the two branches are concatenated to obtain the final low-frequency information $low_f$ and high-frequency information $high_f$. As shown in Fig. 3, the AFD module uses two branches to extract contrast-aware features with different granularity. Here are the reasons for this design. If we consider the frequency decomposition as a Gaussian filter, the sizes of the dilated convolution kernels of the different branches can be regarded as the sizes of the different Gaussian kernels in the Gaussian filter. In Gaussian filtering, the larger the Gaussian kernel is, the more blurring of the image after filtering. However, it is not the case that a larger Gaussian kernel is better. A too large Gaussian kernel will not only filter out the noise, but also smooth out the useful information in the image. Therefore, we need to simultaneously consider both the noise suppression effect and the preservation of useful content information when designing the dilated rate of the dilated convolution in different branches. Specific parameter settings are given in “Experiments” section. The frequency features of of different granularity and the features $x_{dn}$ extracted by the decoder are concatenated to increase the receptive field^39^ to obtain more global information^40^, thereby improving the ability of network detail recovery. Subsequently, we use the channel attention mechanism to capture the relationship between different channels. Specifically, the concatenated features are then passed through a SE^41^ module to obtain a scaling vector *v* and multiplied with it to re-weight the importance of different channels. By adjusting the weight templates of different branches, the strategy of dynamic selection of main components mechanism^42,43^ is realized.

### Analysis of CNN features

In this subsection, we considers the interrelationships between the frequency features and hierarchical features of a network, helping mine the low frequency and high frequency information of the optimal network layer. In the U-Net network, the feature scale of the encoding branch gradually decreases with the increase of depth. Inspired by Refs.^21,44^, the layers of different depths of the U-Net get different feature characteristics. The receptive field of high-scale layer is small, mainly including geometric details such as local details and noise; the receptive field of low-scale layer is large, mainly including semantic information such as background and illumination.

**Figure 4:**
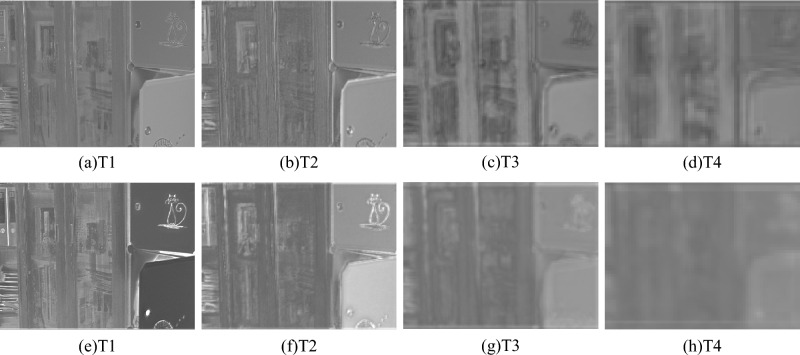
Extracting visual feature maps of different layers based on pre-trained U-Net. The first row is the result of normal U-Net output, and the second row is the result of embedding AFD module on top of it. The high-frequency information extracted at larger scales (i.e. T1 and T2) mainly contains local detail information; the low-frequency information at smaller scales (i.e. *T*3 and *T*4) mainly contains global information.

To verify the above view, We perform frequency analysis by using pre-trained U-Net (i.e. with and without AFD module) networks with frequency information extracted from different scales. For the design comparison experiments, we add AFD modules to the T1 and T2 layers of the U-Net benchmark model to extract high-frequency information and to extract low-frequency information in the T3 and T4 layers. Both models are trained on the public LOL-V1^16^ training set and analyzed on the LOL-V1 test set. Then, we use the total variance (TV) loss^45^ to compute the local gradient values which can be considered as the local details. The larger its value, the more high-frequency information; the smaller its value, the more low-frequency information. To facilitate comparison, we normalize the TV loss distribution. As shown in Fig. 4 and Table 1, the low-frequency information distribution is closer to the low-scale space (i.e. T3 and T4), while the high-frequency information distribution is closer to the high-scale space (i.e. T1 and T2). As can be seen from Table 1, AFD module can improves frequency distribution in different scale spaces. Figure 4 depicts a visual comparison, which corrobarates the numerical result. Without the AFD module, the feature map has information overlap and interference, and the low and high-frequency features are not better represented. The noise and gradient information is amplified in the low-scale space, and the expected detail information is over-smoothed in the high-scale space. In addition, if we recover the low-frequency information better in the low-scale space and suppress the noise, it will also help the network to recover the high-frequency details in the high-scale space. Therefore, we extract the low-frequency information $low_f$ in the low-scale space (i.e. T3 and T4) and extract the high-frequency information $high_f$ in the high-scale layer (i.e. T1 and T2), so that the most abundant and important semantics contained in different scale spaces can be effectively utilized. In the following experiments, we use this setup for all analyses.

**Table 1:** Total variance (TV) loss for different layers of CNN features.

Features	T1 layer	T2 layer	T3 layer	T4 layer
U-Net	0.3913	0.2238	0.2355	0.1493
U-Net with AFD module	0.3946	0.4526	0.0665	0.0863

## Loss function

**Figure 5:**
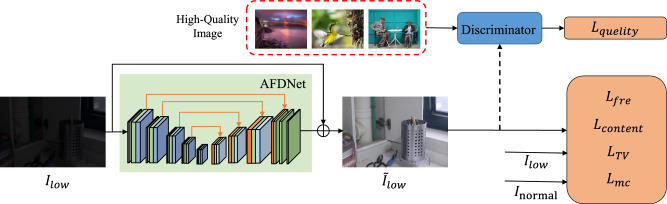
The flowchart of the adversarial generative learning.

To produce results with good reconstructed detail and guarantee satisfactory contrast and color distribution visually, we proposed a comprehensive loss function to train the network. Figure 5 shows the flowchart of the model with loss functions.

We have a good balance of perceptual quality and fidelity in training the model via adversarial learning. The proposed AFDNet is worked as the generator, and the adversarial generative network model is formed with the designed discriminator. The discriminator CNN consists of seven convolutional layers each followed by a LeakyReLU. The seven convolutional layers, using kernel size 4 $\times$ 4, stride 1, with kernel numbers of 64, 128, 256, 512, 512, 512, and 1 respectively. A sigmoidal activation function is applied to the outputs of the last fullyconnected layer and produces a probability of the input image with high-quality. Note that this is only a part of total loss function, and the overall loss consists of the following five components.

### Frequency loss

Considering that AFDNet subdivides the image into low-frequency and high-frequency components, we design a novel frequency loss function to help the network recover more details with different frequency layers. The enhanced image is transformed into the frequency domain through the FFT, and after low-frequency filtering and high-frequency filtering respectively. The inverse FFT returns the low and high frequency domain to the image space. Wasserstein distance^46^ is used to minimize the difference between the low-frequency and high-frequency information of the enhanced and target images.

6 $$\begin{aligned} L_{fre} = \frac{1}{N^2}\sum _{i=low}^{high} inf_{\gamma \sim \prod ({\tilde{I}}_{low}^i,I_{high}^i)}E_{(x,y)\sim [{\mid \mid {\tilde{I}}_{low}^i-I_{normal}^i\mid \mid }]}, \end{aligned}$$

where ${\tilde{I}}_{low}$, $I_{normal}$ and *N* are the enhanced image, ground truth and training batch size, respectively. $\prod ({\tilde{I}}_{low}^i,I_{high}^i)$ represents the set of all possible joint distributions of the combined distributions ${\tilde{I}}_{low}^i$ and $I_ {High}^i$. The formula after *inf* is to sample the sample pair $({\tilde{I}}_{low}^i, I_{high}^i)\sim \gamma$ from the fixed joint distribution $\gamma$, and calculate the expected value of the distance $E_{(x,y)\sim [{\mid \mid {\tilde{I}}_{low}^i-I_{normal}^i\mid \mid }]}$ of this sample pair. *inf* denotes taking the maximum lower bound of the expected value.

### Content loss

Our content loss ($L_{content}$) contains two parts: reconstruction loss ($L_{rec}$), and a perceptual loss ($L_{vgg}$). We use the L1 loss as the reconstruction loss, producing an enhanced image that is closer to the target image. Inspired by Refs.^47–49^, we use perceptual loss to calculate the VGG feature Euclidean distance between the enhanced image and the target image, encouraging them to have similar feature representations.

7 $$\begin{aligned} L_{content}= & {} L_{rec}+L_{vgg}, \end{aligned}$$

8 $$\begin{aligned} L_{rec}= & {} \frac{1}{N}\sum _{i=1}^{N}\mid {\tilde{I}}_{low}-I_{normal}\mid +\frac{1}{CWH}{\mid \mid {\tilde{I}}_{low}-I_{normal}\mid \mid }_2^2, \end{aligned}$$

9 $$\begin{aligned} L_{vgg}= & {} \frac{1}{C_{i,j}W_{i,j}H_{i,j}} {\mid \mid \phi _{i,j}({\tilde{I}}_{low})-\phi _{i,j}(I_{normal})\mid \mid }_2^2, \end{aligned}$$

where *C*, *H* and *W* are the dimensions of the enhanced image${{\tilde{I}}}_{low}$. $C_{i,j}$, $W_{i,j}$ and $H_{i,j}$ denotes the number, height and width of the feature maps. $\phi _{i,j}$ is the process of extracting deep features from the VGG-16^50^ network pretrained on ImageNet. *i* represents its *i*-th Max pooling, and *j* represents its *j*-th convolutional layer after its *i*-th Max pooling layer, *i* and *j* are set to 5 and 1 here.

### Adversarial loss

Considering that frequency loss and content loss can easily make the network limited by high fidelity, which is not alway well aligned to human visual perception. To solve this problem, we introduce an adversarial loss obtained by an adversarial generative network. Here, the adversarial generative network consists of AFDNet and a discriminator, in which the discriminator is used to predict whether the input image is of high quality or not. As shown in the Fig. 5, we use high-quality images selected from the aesthetic visual analysis (AVA)^51^ dataset based on MOS values as a perceptual guide. Formally, We define the adversarial loss $L_{adv}$ with the conception of cross-entropy:

10 $$\begin{aligned} L_{adv}=-logD(Y)-logD(1-{\tilde{I}}_{low}), \end{aligned}$$

where ${\tilde{I}}_{low}$ is the output enhanced image of AFDNet and Y denotes the unpaired random high quality image.

### Mutual consistency loss

As the mutual consistency loss^18^ can solve the degradation problem in the luminance map, thus the edge information is strengthened and the smooth surface is uniform. Therefore, we introduce it for network training to guarantee mutual consistency between the enhanced image and the input image. The mutual consistency loss $L_{mc}$ is defined as:

11 $$\begin{aligned} L_{mc}= & {} {\mid M\cdot exp(-c \times M)\mid }, \end{aligned}$$

12 $$\begin{aligned} M= & {} {( \nabla {\tilde{I}}_{low})}^2 +{( \nabla I_{low})}^2, \end{aligned}$$

where $\nabla$ stands for the first order derivative operator containing $\nabla _x$(horizontal) and $\nabla _y$(vertical) directions. *c* is a parameter that controls the shape of the function, called the penalty factor. The smaller the penalty factor *c*, the weaker the nonlinearity between *M* and $L_{mc}$ ; the larger the value of the penalty factor *c*, the stronger the nonlinearity.

### Total variational loss

In order to reduce the noise, the total variational (TV) loss^45^ is adopted to ensure the spatial smoothness of the enhanced image. The total variation loss $L_{TV}$ is defined as:

13 $$\begin{aligned} L_{TV}= \frac{1}{CWH}{(\mid \nabla _x{{{\tilde{I}}}_{low}}\mid +\mid \nabla _y{{{\tilde{I}}}_{low}}\mid )}^2. \end{aligned}$$

### Total loss

The comprehensive loss function for AFDNet is weighted sum up above losses as follows:

14 $$\begin{aligned} L_{total}=\lambda _1{L_{fre}} +\lambda _2 {L_{content}} + \lambda _3{L_{quality}} + \lambda _4{L_{mc}} + \lambda _5{L_{TV}}, \end{aligned}$$

where $\lambda _1$, $\lambda _2$, $\lambda _3$, $\lambda _4$ and $\lambda _5$ are weighting parameters.

## Experiments

### Experimental setting

We have implemented the proposed model in the in PyTorch with CUDA acceleration. The LOL-V1 dataset^16^ is used as training set, which including 485 pairs of real low/normal-light images which captured by changing the camera’s exposure time and ISO, each PNG image size is 400 $\times$ 600. During training we randomly crop and flip the input data horizontally. Using the Adam^52^ optimizer, the learning rate is set to 1e−4 for the first 200 epochs, and decays linearly to 0 for the subsequent 200 eporchs. The batch size N is 32, and the learnable amplification parameter $\alpha ^i$ is set to 1 at the beginning of training. The dilated rates $d_1^1$, $d_1^2$, $d_2^1$ and $d_2^2$ of different branches are set to 1, 6, 1, and 12 respectively. The penalty factor c is set to 10. $\lambda _1$, $\lambda _2$, $\lambda _3$, $\lambda _4$ and $\lambda _5$ are set to 5, 1, 0.5, 5, and 1. The whole training takes 5 h on 4 Nvidia 1080Ti GPUs.

### Evaluation datasets and metrics

We evaluate AFDNet on widely public datasets, including LOL (V1 & V2)^16^, DICM^2^, LIME^53^, MEF^54^, and NPE^55^. These test sets are all downloaded from the evaluation set provided by Ref.^13^. We evaluate the performance of different methods from different perspectives. The evaluation metrics include reference metrics and no-reference metrics, including: PSNR, SSIM^56^, MSE, AB^57^, LPIPS^58^, NIQE^59^. Among them, PSNR, MSE, and SSIM are widely used IQA metrics in low-level vision tasks to evaluate the similarity between enhanced result and reference images. Learning Perceptual Image Patch Similarity (LPIPS) is also known as “perceptual loss”. Compared with traditional indicators, the LPIPS is estimated by calculating the distance metric between features, which is more suitable for human visual perception of texture. A lower LPIPS value indicates a higher perceptual similarity of the enhanced image to the corresponding groundtruth. The Average Brightness(AB) calculates the brightness of the enhanced image. Natural Image Quality Evaluator (NIQE) evaluates the quality of the enhanced image based on human perceptual similarity. The lower the value, the closer the enhanced image is to the natural. The higher the PSNR, SSIM, AB value, the better the quality, and the opposite for MSE, LPIPS, NIQE. The superiority of our method is demonstrated by quantitative and qualitative comparison with the state-of-the-art methods currently available with public code.

### LOL (V1 & V2) dataset variational

**Figure 6:**
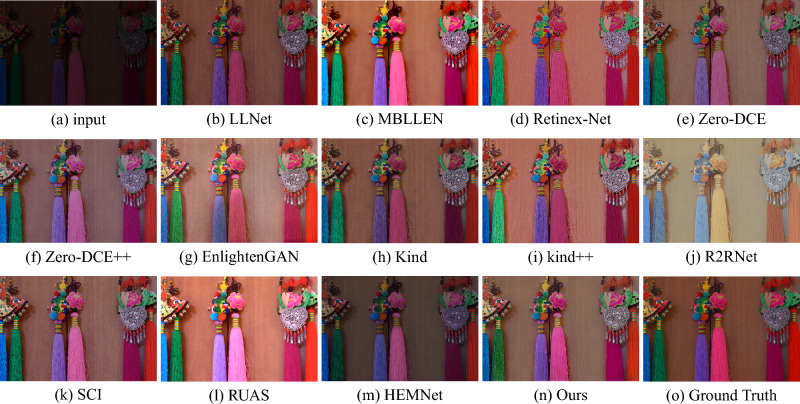
Visual comparison with state-of-the-art low-light image enhancement methods on the LOL-v1 dataset.

**Figure 7:**
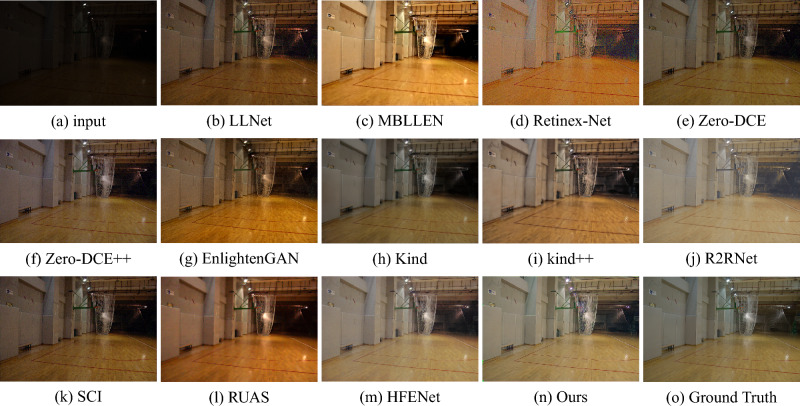
Visual comparison with state-of-the-art low-light image enhancement methods on the LOL-v2 dataset.

The LOL (V1 & V2) test set is collected by controlling for exposure and ISO, and each low-light image has a corresponding normal-light image to calculate quantitative metrics. There are 15 pairs of images in LOL-v1 and 100 pairs of images in LOL-v2. For overall comparison, we select 12 most representative state-of-the-art methods, including LLNet^14^, MBLLEN^17^, Retinex-Net^16^, Zero-DCE^12^, Zero-DCE++^20^, EnlightenGAN^13^, Kind^18^, Kind++^19^, R2RNet^60^, SCI^29^, RUAS^28^ and HFMNet^21^. The evaluation results of our method and other state-of-the-art methods on the LOL (V1 & V2) dataset are presented in Table 2. Through quantitative comparison, AFDNet achieves better performance in all with and without reference metrics, especially higher PSNR and SSIM and lower LPIPS. Representative results are visually shown in Figs. 6 and 7. It clear that all the previous methods can effectively improve the brightness and contrast, but none of the previous methods can well restore global illumination and structures. Among them, the resulting images of LLNet, Zero-DCE and Zero-DCE++ have problems of brightness, low contrast and blurred images. MBLLEN, Retinex-Net and EnlightenGAN can produce better visual effects, but all cause false information and amplify noise in dark areas. In comparison, the enhanced images obtained by Kind and Kind++ are more natural, but the enhancement of contrast and brightness is slightly insufficient. R2RNet, SCI, RUAS and HFENet can improve the local and global contrast better, but still have the problem of missing details. Comparatively, AFDNet achieves good perceptual visual quality with sharp details, uniform color distribution, and better noise suppression.

**Table 2:** Quantitative comparison on LOL (V1 & V2) dataset in terms of PSNR, SSIM, MSE, AB, LPIPS, and NIQE.

Method	LOL-V1						LOL-V2					
	PSNR$\uparrow$	SSIM$\uparrow$	MSE$\downarrow$	AB $\uparrow$	LPIPS$\downarrow$	NIQE$\downarrow$	PSNR$\uparrow$	SSIM$\uparrow$	MSE$\downarrow$	AB $\uparrow$	LPIPS$\downarrow$	NIQE$\downarrow$
LLNet^14^	28.1241	0.5129	101.5715	64.3940	0.3491	8.0910	27.9700	0.5269	104.5375	61.8057	0.3477	8.2731
MBLLEN^17^	28.0733	0.7863	103.3022	100.9130	0.2111	4.3592	27.9410	0.6423	104.7113	80.2747	0.2737	4.8441
Retinex-Net^16^	28.0565	0.4191	101.8668	107.7655	0.4443	8.8792	27.9991	0.4006	103.2850	101.5877	0.5429	9.4274
Zero-DCE^12^	27.8289	0.6626	108.6866	72.0175	0.3142	7.7667	28.1533	0.5735	101.2444	65.0908	0.3111	8.0578
Zero-DCE++^20^	28.0184	0.5619	103.8069	73.5041	0.3208	7.8963	28.1504	0.5718	101.3338	64.6287	0.3126	8.0448
EnlightenGAN^13^	27.8051	0.7319	108.6318	94.7671	0.2968	6.4889	28.1550	0.6754	99.7077	82.7959	0.3088	5.0888
Kind^18^	27.9912	0.7748	104.1301	103.2690	0.1641	4.7101	28.0865	0.7145	101.8748	88.9531	0.3770	5.5608
Kind++^19^	28.1693	0.7696	102.6426	109.1153	0.1853	4.7663	28.1095	0.7695	100.9257	*107.8107*	0.2165	4.6714
R2RNet^60^	28.1359	0.74317	100.6120	**124.3166**	0.2851	*4.0877*	28.0023	0.7747	*84.6253*	121.3354	0.2724	4.9576
SCI^29^	27.9048	0.5219	105.9507	69.2669	0.3393	7.8767	27.9600	0.5335	104.9082	63.76475	0.3079	8.0461
RUAS^28^	28.0313	0.4996	102.7765	94.13435	0.2701	6.3401	27.9219	0.4875	105.2823	85.2675	0.3097	6.5331
HFMNet^21^	*28.5593*	*0.8147*	*93.2648*	112.6408	**0.1268**	4.5616	*28.8586*	*0.8273*	88.1210	104.6577	*0.1406*	*4.4929*
Ours	**28.9181**	**0.8238**	**88.9780**	*117.1915*	*0.1436*	**4.0775**	**29.3200**	**0.8373**	**79.1373**	100.2312	**0.1397**	**4.3192**

The up or down arrows represent the metrics go direct in good. best and second best results are marked in bold and italic respectively.

### Ablation study

**Figure 8:**
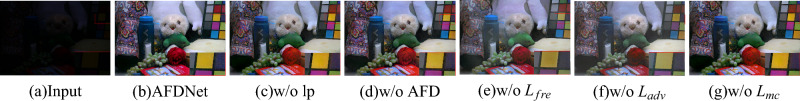
Ablation study of the contribution of Laplace pyramid (lp), AFD module (AFD) and each loss (frequency loss $L_{fre}$, adversarial loss $L_{adv}$, mutual consistency loss $L_{mc}$). Note the edge details and overall illumination of the image.

To explore the effectiveness of laplace pyramid, adaptive frequency decomposition (AFD) module and loss function settings, we conduct experiments by removing the laplace pyramid, removing the AFD module and removing different loss functions, respectively. The LOL-V1 dataset is also used to calculate different enhancement result metrics. As shown in Table 3, removing the Laplace pyramid leads to performance degradation, because the Laplace pyramid provides richer and more realistic texture features at multiple scales to guide the encoder–decoder architecture recovery image details. Similarly, removing the AFD module also leads to performance degradation, because the AFD module can guide the enhancement network to extract low-frequency information for image noise suppression and high-frequency information for detail recovery, resulting in better performance. This verifies the effectiveness of AFD module in extracting useful features and suppressing harmful features in optimal scale features spaces. An additional visual comparison is shown in Fig. 8, the model without the laplace pyramid results in blur detail and the model without AFD module results in color deviation and obstinate noise. In contrast, the model with both Laplace pyramid and AFD module contributes to a better visual quality.

**Table 3:** Ablation studies.

Conditions	PSNR$\uparrow$	SSIM$\uparrow$	MSE$\downarrow$
1. AFDNet	**28.9181**	**0.8238**	**86.4054**
2. w/o Laplace pyramid	28.6711	0.8031	91.2953
3. w/o AFD module	28.1433	0.7922	94.1497
4. w/o frequency loss	28.7616	0.8010	88.9780
5. w/o adversarial loss	28.5061	0.8125	86.6024
6. w/o mutual consistency loss	28.7075	0.8031	90.3771

This table reports the performance under each condition based on the LOL-V1 dataset. In this table, “w/o” means without.

The best results are highlighted in bold.

Further, we remove the frequency loss, adversarial loss and the mutual consistency loss separately for experiments, and the experiments show that the removal of the frequency loss and the mutual consistency loss leads to the performance degradation. The result of the model without using frequency loss is decreased by 0.0228 dB and 0.1565 in terms of the PSNR and SSIM, and it is increased by 2.5726 in terms of the MSE. The result of the model without using adversarial loss is decreased by 0.0113 dB and 0.4120 in terms of the PSNR and SSIM, and it is increased by 0.1970 in terms of the MSE. The result of the model without using mutual consistency loss is decreased by 0.2106 dB and 0.0207 in terms of the PSNR and SSIM, and it is increased by 3.9717 in terms of the MSE. These results demonstrated the effectiveness of our loss function setting.

### No-referenced image quality assessment

To fully evaluate AFDNet, we perform quantitative comparisons on four publicly available natural low-light datasets (DICM, NFE, LIME, MEF). Since the above datasets do not have paired reference images, we adopt four reference-free evaluation metrics(NIQE^61^, CEIQ^62^, LOE^63^, DE^61^). The results are shown in Tables 4 and 5.

**Table 4:** NIQE and CEIQ scores on MEF, LIME, NPE, DICM datasets, respectively.

Methods	MEF		LIME		NPE		DICM		AVG	
	NIQE$\downarrow$	CEIQ$\uparrow$	NIQE$\downarrow$	CEIQ$\uparrow$	NIQE$\downarrow$	CEIQ$\uparrow$	NIQE$\downarrow$	CEIQ$\uparrow$	NIQE$\downarrow$	CEIQ$\uparrow$
MBLLEN^17^	3.6940	3.0532	5.0699	3.2503	4.6265	3.2160	4.5147	3.3078	4.2059	3.2068
Retinex-Net^16^	4.4972	3.0088	4.2942	3.2193	4.8011	3.0312	5.6886	3.2260	4.8749	3.1213
Kind^18^	3.8612	2.8506	3.6715	3.1284	4.3540	3.0963	4.6410	2.7652	4.1098	2.9601
R2RNet^18^	4.8074	2.8936	4.5357	3.3553	4.1313	3.2321	4.8074	3.1149	4.3558	3.1490
SCI^29^	4.4604	2.4845	4.4898	3.1068	3.8894	3.1223	3.6408	2.7286	4.0085	2.8606
RUAS^28^	5.4656	2.6000	5.0795	2.4321	4.9412	2.5468	4.5572	2.7652	4.8308	2.5860
EnlightenGAN^13^	3.5869	2.8820	3.8354	3.3227	3.4953	**3.2995**	2.9186	3.2957	3.2355	3.2000
Zero-DCE^12^	4.0550	2.7637	3.7860	3.2287	3.396	3.0822	2.8249	3.2385	3.4973	3.0783
Zero-DCE++^20^	4.3356	2.6746	4.1951	3.2751	3.5119	3.2037	2.8910	3.2853	3.5327	3.1097
HFMNet^21^	4.0170	**3.1488**	3.8724	3.2423	3.7705	3.2229	3.2384	3.2862	3.5848	3.2250
Kind++^19^	**3.1143**	3.0486	**3.4905**	3.2349	5.0014	3.2153	4.1043	3.2377	3.6611	3.1841
Ours	3.3563	3.1427	4.0499	**3.3661**	**3.2302**	3.2935	**2.8159**	**3.3730**	**3.2118**	**3.2938**

The best results are highlighted in bold.

**Table 5:** LOE and DE scores on MEF, LIME, NPE, DICM datasets, respectively.

Methods	MEF		LIME		NPE		DICM		AVG	
	LOE$\downarrow$	DE$\uparrow$	LOE$\downarrow$	DE$\uparrow$	LOE$\downarrow$	DE$\uparrow$	LOE$\downarrow$	DE$\uparrow$	LOE$\downarrow$	DE$\uparrow$
MBLLEN^17^	256.54	7.14	214.65	7.1770	267.45	7.1461	396.62	7.32	321.32	7.19
Retinex-Net^16^	473.85	6.92	508.73	7.08	849.12	6.82	600.11	7.17	579.59	7.00
Kind^18^	378.41	6.71	302.41	6.91	354.98	6.85	**305.33**	6.28	334.20	6.69
R2RNet^18^	270.55	6.52	177.26	7.31	212.51	7.14	356.05	6.81	393.05	6.94
SCI^29^	389.66	5.53	247.12	6.8741	274.12	6.81	430.83	6.31	374.94	6.38
RUAS^28^	528.40	5.90	387.23	5.93	342.15	5.78	334.52	6.28	467.44	5.97
EnlightenGAN^13^	443.76	6.83	183.76	7.29	208.34	7.22	653.94	7.31	572.83	7.16
Zero-DCE^12^	398.44	6.15	391.00	7.13	652.13	6.86	594.28	7.24	320.38	6.84
Zero-DCE++^20^	265.48	6.25	184.26	7.24	426.45	7.14	365.44	7.29	335.34	6.98
HFMNet^21^	303.39	7.13	231.46	7.22	294.04	7.05	364.52	7.14	309.03	7.14
Kind++^19^	356.21	7.00	198.78	7.15	231.24	7.08	420.87	7.20	377.23	7.11
Ours	**235.67**	**7.23**	**158.05**	**7.43**	**195.41**	**7.37**	358.67	**7.46**	**283.04**	**7.37**

The best results are highlighted in bold.

**Figure 9:**
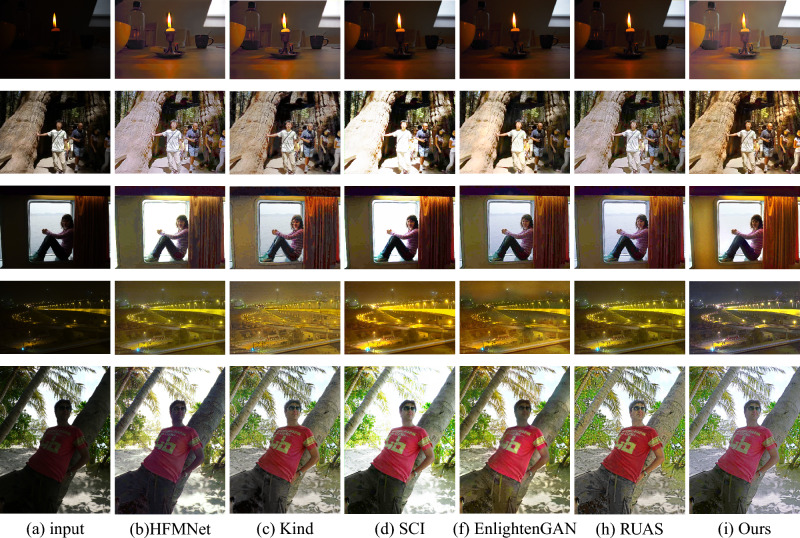
Visual comparison with state-of-the-art low-light image enhancement methods whth various low-light conditions. Row 1–5 display non-uniform illumination, side lighting, backlight, nighttime and high noise scenes.

The lower the values of NIQE and LOE, and the higher the values of CEIQ and DE, indicate that the image is more natural and closer to the normal light image distribution. The results further shows the superiority of AFDNet over other state-of-the-art methods in generating high-quality visual results. In order to verify the enhancement effect of our model under different lighting conditions, we conducted a comprehensive experiments covering a variety of lighting environments. As shown in Fig. 9, row 1–5 display non-uniform illumination, side lighting, backlight, nighttime and high noise scenes. It’s obvious to see that AFDNet achieved more satisfactory visualization results than others, in exposure control, noise suppression, color uniformity, etc.

**Figure 10:**
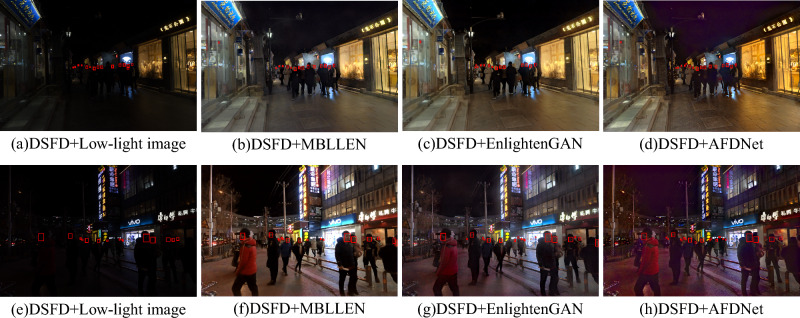
Example of face detection results. We used EnghtenGAN, MBLLEN and our AFDNet as preprocessing steps, and then used DSFD for detection.

**Figure 11:**
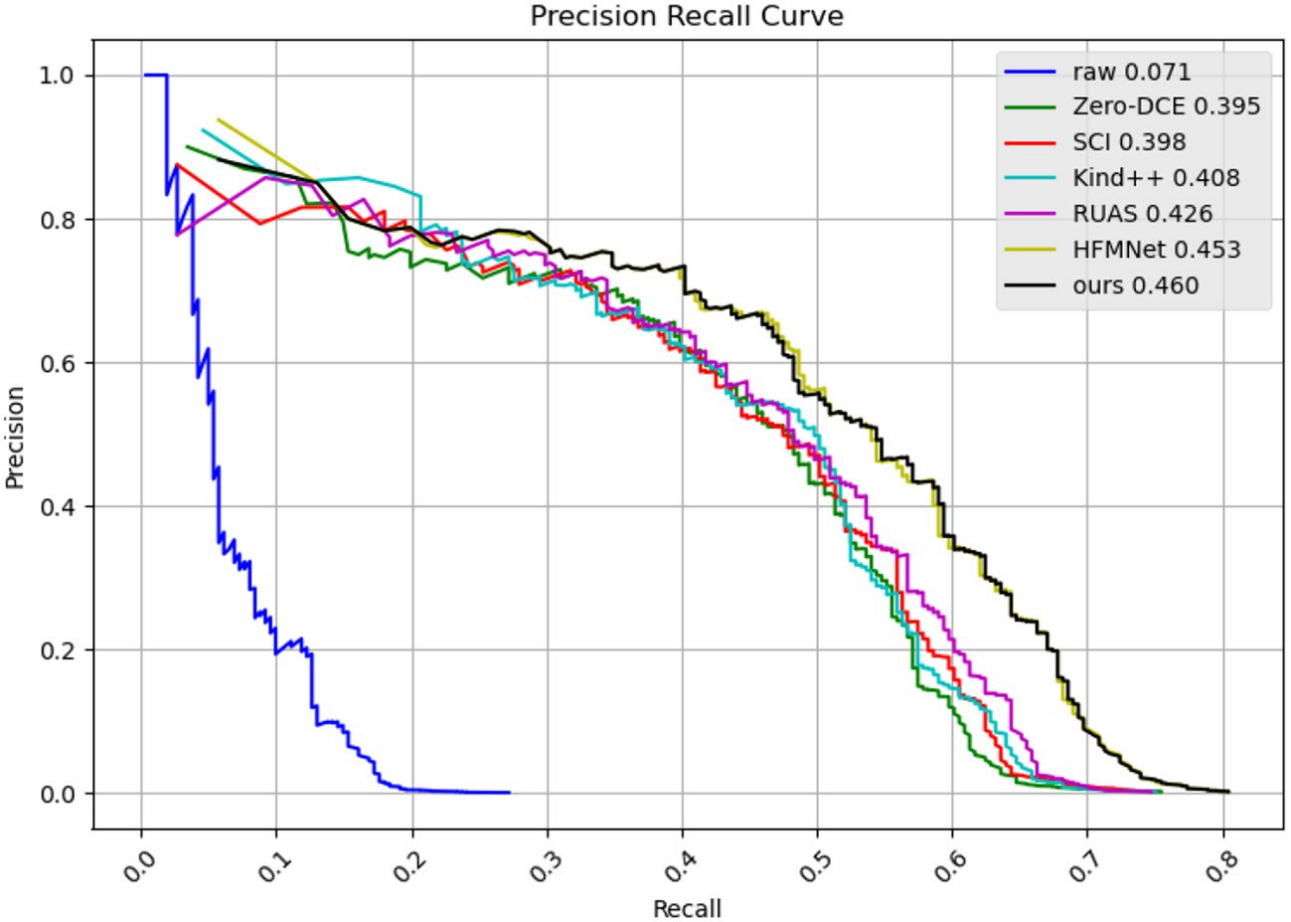
Performance of face detection method DSFD in dark environments. PR curve and AP.

### Pre-processing for improving detection

We investigate face detection performance in low-light conditions. Specifically, we use the DARK FACE dataset^64^, which contains 6100 real-world low-light images captured at night, including 6000 training/validation set images and 100 test set images. Because the annotations of the test set are not publicly available, we randomly select 100 images from the training set for evaluation. A well-performed face detector, Dual Shot Face Detector (DSFD)^65^, is adopted as the baseline model. We integrate different low-light image enhancement methods into DSFD for face detection, and Fig. 11 is the precision–recall (P–R) curve of different methods. In addition, we also compare average precision (AP) by using the evaluation tools provided in the DARK FACE dataset.

As shown in Fig. 11, after image enhancement, the accuracy of DSFD is greatly improved compared with the original unenhanced image. Among different methods, AFDNet and HFMNet perform eminently but AFDNet performs better in both precision and recall. Using our method as preprocessing, the average precision (AP) increases from 7.1 to 46.0%, which demonstrates that AFDNet can improve the performance of computer vision tasks. Two low-light real sceneries images are presented to illustrate the effectiveness of AFDNet. Compared with mainstrem enhancement networks MBLLEN and EnglightenGAN, the visulized images are shown in Fig. 10. AFDNet can significantly improve the image brightness and restore the details in dark areas, which greatly improving the performance of the detector.

## Conclusion

In this research, we proposed a novel Adaptive Frequency Decomposition Network (AFDNet) to enhance low-light images. The proposed network increases the feature width through the Laplacian pyramid, which guides the encoder–decoder to recover image details. An Adaptive Frequency Decomposition (AFD) module is designed to connect encoding and decoding, which can adaptively extract frequency information from optimal scale feature space for detail recovery and image denoising. The end-to-end deep learning method proposed a novel comprehensive loss function to constrain model training, including frequency loss, content loss, adversarial loss, mutual consistency loss, and total variational loss. Among these, the adversarial loss improves the perceptual vidual quality of the image via adversarial learning, so that the enhanced image look more natural and have better visual effects. Additionally, a novel frequency loss function is used to help AFDNet recover more image details. Qualitative and quantitative evaluations on public datasets show that AFDNet has obvious advantages over state-of-the-art methods, and can achieve better visual quality, has unique advantages in detail recovery and noise suppression. It is also confirmed that AFDNet can effectively improve the performance of nighttime face detection. For future directions, we are interested in introducing image segmentation information to low-light image enhancement.

## Data Availability

The datasets generated during and/or analysed during the current study are available from the corresponding author on reasonable request.

## Accession codes

The proposed AFDNet backbone network is available publicly for open accessat AFDNet source.
